# The role of bioactive lipids and eicosanoid metabolites in acute exercise in adults: Insights into human cardiorespiratory fitness

**DOI:** 10.14814/phy2.70671

**Published:** 2025-12-05

**Authors:** Mythri Ambatipudi, Athar Roshandelpoor, J. Sawalla Guseh, Leah B. Kosyakovsky, Mona Alotaibi, Susan Cheng, Mohit Jain, Gregory D. Lewis, Jennifer E. Ho

**Affiliations:** ^1^ CardioVascular Institute and Division of Cardiology, Department of Medicine Beth Israel Deaconess Medical Center Boston Massachusetts USA; ^2^ Cardiology Division, Department of Medicine Massachusetts General Hospital Boston Massachusetts USA; ^3^ Division of Pulmonary and Critical Care and Sleep Medicine University of California San Diego La Jolla California USA; ^4^ Department of Cardiology Smidt Heart Institute, Cedars‐Sinai Medical Center Los Angeles California USA; ^5^ Department of Medicine and Department of Pharmacology University of California San Diego La Jolla California USA; ^6^ Cardiovascular Research Center and Division of Cardiology, Department of Medicine Massachusetts General Hospital Boston Massachusetts USA

**Keywords:** acute exercise, cardiorespiratory fitness, CPET, eicosanoids, metabolomics, peak VO_2_

## Abstract

The molecular mechanisms underlying the salutary effects of exercise remain incompletely understood. Exerkines are signaling molecules with autocrine, paracrine, or endocrine effects released in response to exercise. Specific eicosanoids, small bioactive lipids, act as exerkines. Using a mass spectrometry‐based platform, we assayed eicosanoids and related metabolites at rest and peak exercise in individuals undergoing cardiopulmonary exercise testing (CPET). We examined changes in metabolites with exercise and associations with cardiorespiratory fitness measured by peak VO_2_ using multivariable linear regression. We studied 491 individuals (61% women, mean age 57 ± 15). We found 523 (59%) metabolites that dynamically changed with acute exercise (FDR *q* < 0.05). Of these, 278 (53%) including linoleic acid and arachidonic acid derivatives increased, and 245 (47%) decreased, including trihydroxyoctadecenoic acids (triHOMEs) and omega‐3 fatty acids. For 39 metabolites, the magnitude of exercise‐induced change correlated with peak VO_2_, including omega‐3 and omega‐6 fatty acids and linoleic, palmitic, stearic, and arachidonic acid derivatives. We identified lipid metabolites underlying metabolomic responses to acute exercise that relate directly to cardiorespiratory fitness. Anti‐inflammatory linoleic and arachidonic acid derivatives increased with exercise, while pro‐inflammatory and pro‐atherogenic triHOMEs decreased. Future studies may fully delineate metabolomic contributions to the effects of exercise including chronic exercise training.

## 1 INTRODUCTION

Exercise has wide‐ranging impacts on cardiovascular and overall health, yet the molecular mechanisms underlying the salutary effects of exercise are not well understood. (Neufer et al., 2015) While many studies have focused on the impact of exercise on single organ systems, recent studies have leveraged large‐scale blood molecular profiling methods to understand broader patterns of regulation and potential beneficial mechanisms of exercise via analysis of signaling molecules called “exerkines” (Chow et al., 2022; Nayor et al., 2020; Robbins & Gerszten, 2023). Exerkines are signaling molecules released by tissues including liver, bone, nervous system, adipose, and muscle in response to exercise that exert autocrine, paracrine, or endocrine effects (Robbins & Gerszten, 2023). As such, exerkines may mediate cardioprotective effects of exercise via metabolic alterations, vascular changes, signaling between the heart and other organs, and direct effects on the myocardium (Robbins & Gerszten, 2023).

Among the various classes of exerkines identified to date, lipid‐derived signaling molecules have emerged as particularly interesting candidates given their roles in metabolism and vascular biology. For example, 12,13‐dihydroxy‐9Z‐octadecenoic acid (12,13‐diHOME), one of the most well‐described exerkines, is a bioactive lipid derived from linoleic acid. Prior experimental and human studies have shown that 12,13‐diHOME is released by brown adipose tissue following a bout of moderate‐intensity exercise and harbors multiple beneficial cardiometabolic effects including modulation of insulin sensitivity, greater skeletal muscle fatty acid uptake, and enhanced inotropy and lusitropy (Lynes et al., 2017; Pinckard et al., 2021; Robbins & Gerszten, 2023; Stanford et al., 2018). Similarly, eicosanoids are another class of bioactive lipids derived from arachidonic acid (AA) and other lipid precursors that have diverse physiological effects, including regulation of inflammation. (Quehenberger & Dennis, 2011). Our group and others have previously identified multiple eicosanoid and related oxylipin metabolites relevant to cardiopulmonary disease including heart failure and pulmonary hypertension (Lau et al., 2023; McNeill et al., 2023). Whether these bioactive lipids act similarly as exerkines remains largely unknown.

Finally, cardiorespiratory fitness is an important marker of human health and can be estimated using peak VO_2_, the maximal oxygen consumption attained during an acute bout of exercise (Cade et al., 2018). Peak VO_2_ bears prognostic information across both health and disease, and is often used to risk stratify patients with chronic heart failure (Cade et al., 2018; Swank et al., 2012). We sought to investigate whether acute changes in exerkines are related to fitness as measured by peak VO_2_ to prioritize metabolites most likely to be of biological importance.

In this context, we leveraged a novel mass spectrometry‐based platform to ascertain >250 eicosanoids and related bioactive lipid metabolites to comprehensively assess acute changes with exercise. We hypothesized that acute changes in these lipid metabolites during exercise will lend insights into the molecular architecture and metabolic pathways most relevant to exercise and help elucidate potential bioactive lipids relevant to cardiorespiratory fitness.

## 2 METHODS

### 2.1 Study sample

We studied a sample of 521 individuals with chronic dyspnea who participated in an observational hospital‐based study at Massachusetts General Hospital between 2009 and 2017. All participants underwent clinically indicated cardiopulmonary exercise testing (CPET) with invasive hemodynamic monitoring and had available pulmonary arterial blood samples at resting and peak exercise conditions for metabolite profiling.

We excluded participants with incomplete CPET data and those who did not exercise sufficiently during the CPET (*n* = 5). Out of these participants, we excluded those with prior heart or lung transplants (*n* = 3), adult congenital heart disease (*n* = 4), mitochondrial disease (*n* = 6), pulmonary arterial hypertension (*n* = 3), valvular disease (*n* = 16), or left ventricular ejection fraction <50% (*n* = 3). The final sample included 491 participants. All participants provided written informed consent to participate in the study. The study protocol was approved by the institutional review boards of Beth Israel Deaconess Medical Center and Massachusetts General Hospital and was in accordance with the principles outlined in the Declaration of Helsinki.

### 2.2 Clinical assessment and cardiopulmonary exercise testing

All participants underwent medical history, physical examination, and fasting blood draw. Body mass index (BMI) was defined as weight/height^2^ (kg/m^2^). Participants then underwent CPET with invasive hemodynamic monitoring. In brief, a pulmonary artery catheter was placed through the internal jugular vein, and participants then underwent upright cycle ergometry using a standardized exercise protocol to maximal effort (3 min of unloaded exercise followed by 5–20 watt/minute continuous ramp) (Malhotra et al., 2016). Serial gas exchange and hemodynamic measures were collected both during rest as well as every minute during exercise. Specific gas exchange metrics collected included peak VO_2_, percent predicted VO_2_ (Buchfuhrer et al., 1983), respiratory exchange ratio (RER), and ventilatory efficiency (VE/VCO_2_ slope). Hemodynamic measures included repeated measurements of pulmonary arterial pressure (PAP), pulmonary capillary wedge pressure (PCWP), arterial and venous O_2_ gradient (C[a‐v]O_2_), direct Fick cardiac output (CO), and systolic and diastolic blood pressures (SBP and DBP). From the serial hemodynamic measurements, we calculated PCWP/CO and PAP/CO slopes to assess the diastolic and pulmonary vascular responses to acute exercise, respectively (Ho et al., 2020).

### 2.3 Plasma metabolite profiling

Plasma samples were collected at rest before CPET and at peak exercise (defined as the last minute of exercise during a maximal effort study) from all participants after a minimum of 8 h of fasting and were immediately processed and stored at −80°C. Metabolite profiling of plasma samples was then performed at the University of California, San Diego through directed, non‐targeted liquid chromatography‐mass spectrometry (LC‐MS) along with computational chemical networking of spectral fragmentation patterns (Lagerborg et al., 2019; Watrous et al., 2019). Since freeze–thaw cycles may affect analysis (Villanueva et al., 2005), we focused the analysis on samples with only a single freeze–thaw cycle prior to extraction. Protein precipitation and lipid extraction were performed using cold ethanol containing 20 deuterated oxylipin internal standards, with CUDA included as a post‐extraction control. Chromatographic separation and MS detection were performed on a Thermo Vanquish UPLC coupled to a Thermo QExactive Orbitrap mass spectrometer equipped with a heated electrospray ionization source and collision‐induced dissociation fragmentation. Analyses were performed in negative ion mode using a precursor–fragment ion (MS/MS) acquisition strategy with isotopically labeled internal standards for quantification. Isobaric extraction controls were not used. Chemical networking of spectral fragmentation patterns was used to identify identical mass fragments and similar mass shifts among different fragment peaks, thereby allowing the matching of the mass spectra of unknown compounds to those of known reference compounds (Watrous et al., 2019). A total of 936 eicosanoids and related lipid metabolites were detected through this profiling. For each of the metabolites, below detection threshold values were imputed as 25% of the minimum measured value of that metabolite as described previously (Lau et al., 2023). Metabolites for which >90% of the measurements across all patients were undetectable were excluded from analysis, leaving a total of 885 metabolites for subsequent analysis. A full list of the 51 excluded metabolites can be found in Table S1.

### 2.4 Statistical analyses

Clinical and exercise parameters were summarized for the sample. Metabolite concentrations were natural log‐transformed due to right‐skewed distributions. We examined acute changes using the absolute difference between peak and rest metabolite concentrations (∆MET = peak MET−rest MET) and standardized ∆MET to a mean of 0 and standard deviation of 1. We used paired *t*‐tests to evaluate whether ∆MET indicated significant changes with acute exercise, defined as a false discovery rate (FDR)‐adjusted *q* value <0.05 in order to account for multiple hypothesis testing.

We next examined the association of peak VO_2_ (predictor) with ∆MET (response) using multivariable linear regression models. Models were adjusted for clinical covariates known to affect lipid metabolite biology, including age, sex, BMI, aspirin use, and statin use, as well as plate number to account for batch effects. Significance for peak VO_2_ analyses was defined as an FDR‐adjusted *q* value <0.05. Exploratory analyses additionally accounted for RER as a surrogate for exercise intensity. In secondary analyses, we examined the overlap of ∆MET that was significantly associated with peak VO_2_ with other measures of exercise response using multivariable linear regression, including PCWP/CO slope, PAP/CO slope, peak C[a‐v]O_2_, VE/VCO_2_ slope, delta heart rate between rest and peak exercise (∆HR), delta SBP between rest and peak exercise (∆SBP), and delta stroke volume between rest and peak exercise (∆SV). All models were adjusted for the same covariates as in primary analyses.

In order to identify broader patterns of exercise response, dimensionality reduction techniques were used to visualize the metabolites that demonstrated a significant acute exercise response. Uniform Manifold Approximation and Projection (UMAP) (McInnes et al., 2018) was applied to unscaled ∆MET for the subset of metabolites that demonstrated a significant exercise response (Figure 4b). We used hierarchical clustering and identified three metabolite clusters of exercise response, using the elbow method to determine the optimal cluster number. To investigate clinical correlates of metabolite clusters, we computed cluster scores for each of the 491 participants by multiplying individual beta coefficients from peak VO_2_ analyses by ∆MET for each metabolite included in the score and summing the total. We summarized clinical characteristics by tertiles of cluster score, and examined partial correlation coefficients (adjusted for age and sex) of cluster scores with clinical, exercise characteristics, and cardiometabolic biomarkers including age, sex BMI, peak VO_2_, C[a‐v]O_2_, Homeostatic Model Assessment for Insulin Resistance (HOMA‐IR), C reactive protein (CRP), NT‐proBNP, leptin, adiponectin, resistin, and interleukin 6 (IL6) were calculated. All analyses were performed in R studio using R version 4.2.1.

## 3 RESULTS

We studied 491 patients (61% female, mean age 57 ± 15 years) with LVEF ≥50% who underwent clinically indicated CPET with metabolite profiling at rest and peak exercise. Clinical comorbid conditions were frequent, with mean BMI 29.5 ± 6.5 kg/m^2^, 55% with hypertension, 14% with known clinical heart failure, and 16% with diabetes mellitus. Participants exercised to maximal effort with mean peak RER 1.18 ± 0.12, with many displaying impaired cardiorespiratory fitness (peak VO_2_ 16.7 ± 5.3 mL/kg/min; 78 ± 19% predicted peak VO_2_). Further clinical characteristics along with resting and exercise hemodynamic responses are displayed in Table 1.

**TABLE 1:** Clinical characteristics and exercise parameters of sample.

	Total (*n* = 491)	
Clinical measures		
Age, years	57 (15)	
Female sex, *n* (%)	300 (61%)	
Body mass index, kg/m^2^	29.5 (6.5)	
Diabetes mellitus, *n* (%)	80 (16%)	
Hypertension, *n* (%)	271 (55%)	
Past/Present Smoker, *n* (%)	201 (41%)	
Prior heart Failure, *n* (%)	68 (14%)	
eGFR, mL/min/1.73 m^2^	80 (21)	

CPET measures		
	Resting	Peak exercise
Peak VO_2_, mL/kg/min		16.7 (5.3)
% predicted peak VO_2_, %		78 (19)
Peak RER		1.2 (0.1)
VE/VCO_2_ slope		36.9 (8.6)
Peak work load, W		103 (40)
Peak C[a‐v]O_2_		11.2 (2.1)
Systolic blood pressure, mmHg	136 (31)	186 (30)
Diastolic blood pressure, mmHg	76 (11)	89 (16)
Heart rate, bpm	76 (15)	137 (25)
Fick CO, L/min	5.3 (1.6)	12.2 (3.3)
Stroke volume, mL	71 (22)	90 (24)
PCWP, mmHg	6.6 (3.2)	20.4 (7.8)
Mean PAP, mmHg	16.7 (4.6)	35.5 (9.8)
C[a‐v]O_2_, mL O_2_/100 mL blood	5.7 (1.3)	11.3 (2.1)
PCWP/CO slope, mmHg/L/min		2.3 (2.1)
PAP/CO slope, mmHg/L/min		3.2 (2.7)

*Note*: Values represent mean (standard deviation) unless otherwise noted.

Abbreviations: CO, cardiac output; eGFR, estimated glomerular filtration rate; PAP, pulmonary arterial pressure; PCWP, pulmonary capillary wedge pressure; RER, respiratory exchange ratio.

### 3.1 Dynamic changes in lipid metabolites with acute exercise

**FIGURE 1:**
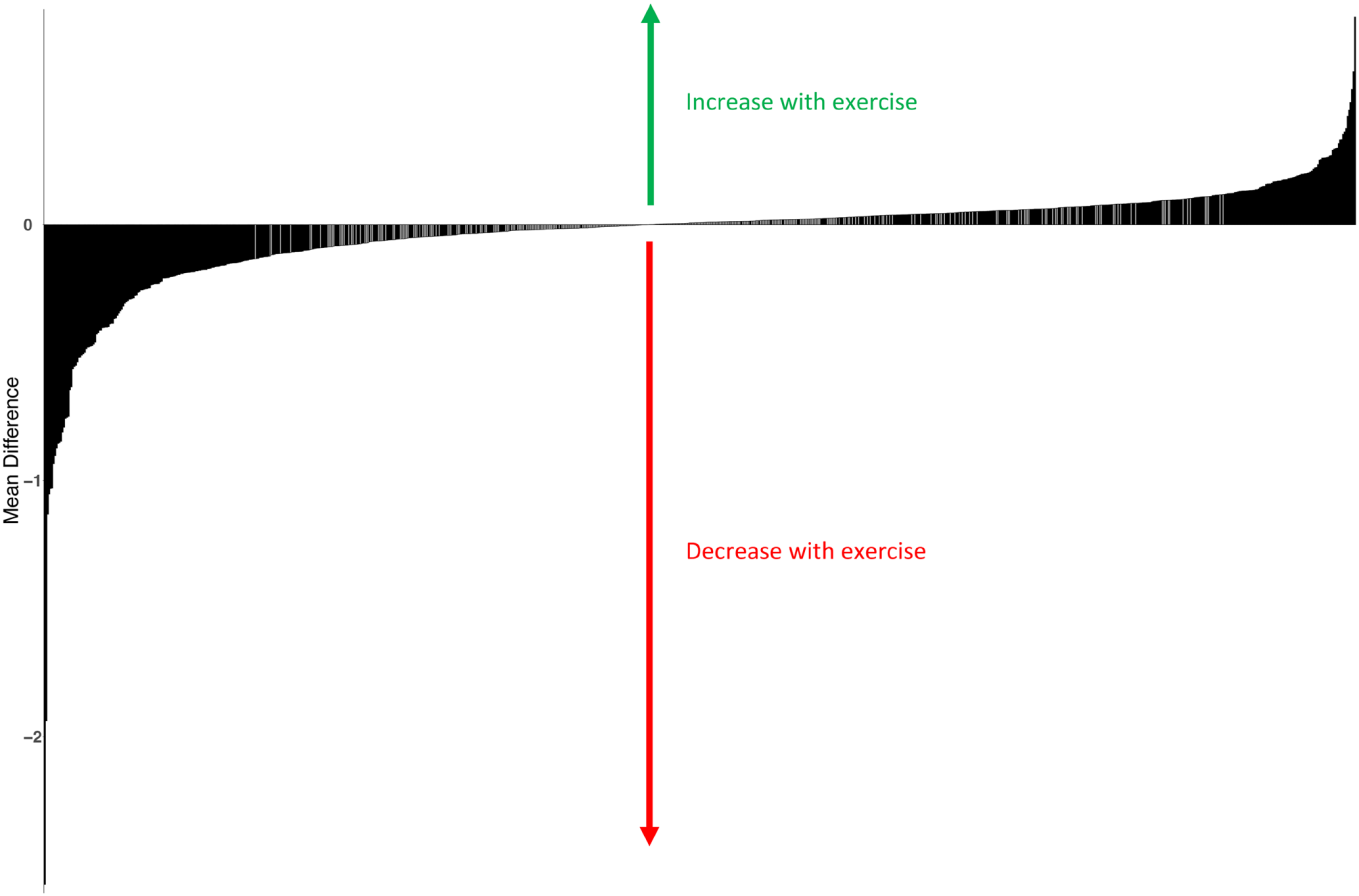
Mean difference between peak exercise and rest of 885 eicosanoids and related lipid metabolites. Metabolites with significant exercise gradients (523/885, *q* < 0.05) are shown in black while those without are shown in gray.

Of the 885 eicosanoid and related lipid metabolites, 523 (59%) demonstrated a significant change with acute exercise (FDR *q* < 0.05 for all, Figure 1). Of these significant metabolites, 245 (47%) decreased and 278 (53%) increased with exercise. Top metabolites with known putative identities are displayed in Table 2, with full results in Table S2. The top metabolites with known molecular identities that increased with exercise included the linoleic acid derivatives 13‐hydroperoxyoctadecatrienoic acid (13‐HpOTrE), which showed a mean difference between peak exercise and rest of 0.13 ± 0.01 SD units, and hydroperoxyoctadecadienoic acids (13‐HpODE/9‐HpODE), which showed a mean difference between peak exercise and rest of 0.10 ± 0.01 SD units. Of note, the well‐studied exerkine 12,13‐diHOME was also among the lipid metabolites that increased with exercise, with a mean difference of 0.04 ± 0.01 SD units. Other metabolites that increased with exercise included AA derivatives (11,12‐EET and 15‐HpETE). In contrast, trihydroxyoctadecenoic acids (TriHOMEs) decreased the most with acute exercise, including top hit 9,10,11‐Trihydroxyoctadec‐12‐enoic acid, which decreased by 1.13 ± 0.07 SD units. Other metabolites that decreased included polyunsaturated fatty acids (adrenic acid) and omega‐3 fatty acids (eicosapentaenoic acid, EPA; docosahexaenoic acid, DHA).

**TABLE 2:** Top eicosanoids and related lipid metabolites with known molecular identity from each of the 3 clusters that display dynamic changes with acute exercise.

Putative metabolite ID	m/z	Retention time, min	Cluster	Mean difference	SE	*p*	FDR *q*
Increases with exercise							
13‐HpOTrE	309.2076	3.786333	1	0.13	0.01	5.6E‐42	6.4E‐41
13‐HpODE/9‐HpODE	311.2231	4.335166	1	0.10	0.01	4.4E‐33	4.0E‐32
20‐COOH‐AA	333.2075	3.804833	1	0.09	0.01	3.1E‐29	2.6E‐28
11,12‐EET	319.228	5.266333	1	0.08	0.01	1.4E‐28	1.2E‐27
15‐HpETE	335.2231	4.230333	1	0.07	0.01	9.3E‐25	6.8E‐24
Decreases with exercise							
9,10,11‐Trihydroxyoctadec‐12‐enoic acid	411.2365	2.824333	3	−1.13	0.07	7.1E‐50	1.1E‐48
6,9,10‐Trihydroxyoctadec‐7‐enoic acid	411.2366	2.380333	3	−0.87	0.04	2.2E‐76	8.6E‐75
8,9,10‐Trihydroxyoctadec‐6‐enoic acid	411.2359	2.479	3	−0.76	0.06	4.3E‐32	3.8E‐31
5,8,11‐Trihydroxyoctadec‐9‐enoic acid	411.2303	2.281667	3	−0.75	0.07	2.0E‐26	1.5E‐25
FA(18:2;O2) isomer 1; Possibly a DiHODE	393.2282	3.6075	3	−0.55	0.05	6.8E‐86	4.0E‐84
FA(18:2;O2) isomer 2; Possibly a DiHODE	393.2267	3.521167	3	−0.52	0.02	1.4E‐26	1.1E‐25
FA(18:2;O2) isomer 3; Possibly a DiHODE	393.226	3.564333	3	−0.47	0.02	9.2E‐75	3.3E‐73
FA(22:4) isomer 1	331.2649	6.610667	2	−0.43	0.02	1.2E‐100	1.3E‐98
Adrenic acid	331.2595	6.508917	2	−0.40	0.02	4.2E‐89	2.9E‐87
6‐HOME	379.2468	5.846	2	−0.39	0.02	4.9E‐87	3.1E‐85
Oxononanoic/Hydroxynonenoic acid	343.2126	1.554	3	−0.35	0.03	1.4E‐23	9.6E‐23
EPA	301.2173	6.240667	2	−0.26	0.01	5.0E‐97	4.9E‐95
Hydroxyhexadecanoic acid	353.2314	4.3845	2	−0.23	0.01	1.7E‐68	4.0E‐67
13‐oxoODE	293.2125	4.483167	2	−0.22	0.01	9.9E‐80	4.2E‐78
DHA	327.2328	6.401	2	−0.20	0.01	1.1E‐73	3.6E‐72
Arachidonic acid	303.233	6.438	2	−0.19	0.01	3.8E‐77	1.5E‐75

*Note*: Mean difference represents peak minus rest concentration and is displayed in standard deviation units.

Abbreviations: 11,12‐EET, 11,12‐epoxyeicosatrienoic acid; 13‐HpODE, 13S‐hydroperoxy‐9Z,11E‐octadecadienoic acid; 13‐HpOTrE, 13‐hydroperoxy‐9,11E,15Z‐octadecatrienoic acid; 13‐oxoODE, 13‐keto‐9Z,11E‐octadecadienoic acid; 15‐HpETE, 15S‐hydroperoxy‐5Z,8Z,11Z,13E‐eicosatetraenoic acid; 20‐COOH‐AA, 20‐carboxyarachidonic acid; 6‐HOME, 6‐hydroxyoctadecenoic acid; 9‐HpODE, 9R‐hydroperoxy‐10E,12Z‐octadecadienoic acid; DHA, 4Z,7Z,10Z,13Z,16Z,19Z‐docosahexaenoic acid; EPA, 5Z,8Z,11Z,14Z,17Z‐eicosapentaenoic acid; m/z, mass‐to‐charge ratio.

### 3.2 Association of Acute Lipid Metabolite Exercise Responses with peak VO_2_

**FIGURE 2:**
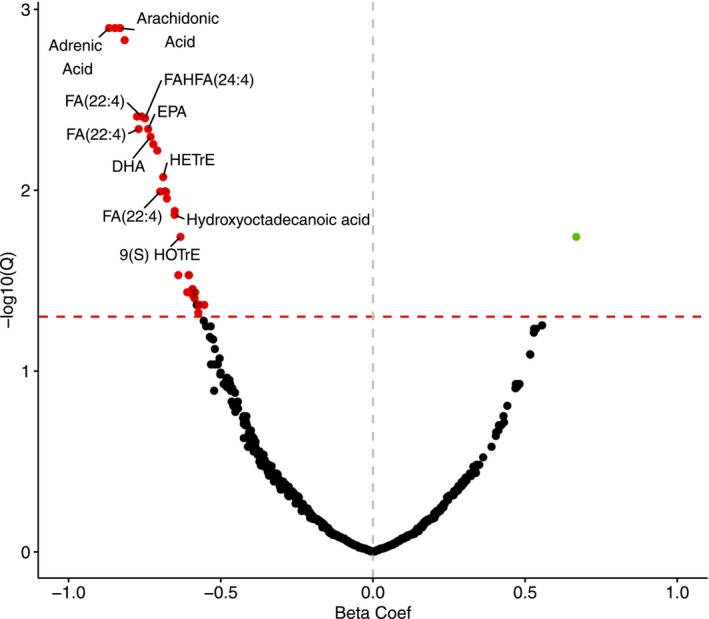
Volcano plot showing associations of ∆MET with acute exercise and peak VO_2_. Beta coefficients represent change in peak VO_2_ in ml/kg/min per 1‐SD change in ∆MET in multivariable‐adjusted models. Significant associations (FDR adjusted *q* value <0.05) were found for 39 eicosanoids and related lipid metabolites, out of which three had positive ∆MET (green) while 34 had negative ∆MET (red). Models adjusted for age, sex, BMI, aspirin use, statin use, and plate number.

Of 885 lipid metabolites assayed, 39 (4.4%) demonstrated an association between the magnitude of acute change during exercise (∆MET) and overall cardiorespiratory fitness as measured by peak VO_2_ (FDR *q* < 0.05 for all, Table 3). Of these 39 metabolites, 34 (87.2%) demonstrated an acute decrease with exercise (negative ∆MET), with a greater exercise excursion in ∆MET associated with a higher peak VO_2_ (Table 3, Figure 2). For example, adrenic acid decreased with acute exercise, with a greater magnitude of decrease associated with higher peak VO_2_ (β = −0.87, s.e. 0.18, *p* = 1.8 × 10^−6^). Other top hits with known molecular identities that similarly demonstrated negative ∆MET, with a greater decrease associated with better cardiorespiratory fitness, included omega‐6 fatty acids (AA and adrenic acid), omega‐3 fatty acids (EPA and DHA), fatty acids (FA(22:4)), hydroxyoctadecanoic acid, hydroxyhexadecanoic acid, and fatty acid esters of hydroxy fatty acid (FAHFA(24:4)). Specific lipid metabolite classes included dihomo‐gamma linoleic acid (DGLA) derivatives (hydroxyeicosatetraenoic acid (HETrE)), and linoleic acid derivatives (9(S)‐hydroxyoctadecatrienoic acid (9(S)‐HOTrE) and 13‐oxooctadecadienoic acid (13‐oxoODE)).

**TABLE 3:** Eicosanoids and related lipid metabolites significantly associated with Peak VO_2_.

Putative metabolite ID	m/z	Retention time, min	Cluster	∆met	Beta Coef	SE	*p*	FDR *q*
Adrenic acid	331.2595	6.508917	2	↓	−0.87	0.18	1.8E‐06	1.3E‐03
	293.2125	5.439	2	↓	−0.85	0.18	3.0E‐06	1.3E‐03
Arachidonic acid	303.233	6.438	2	↓	−0.83	0.18	4.3E‐06	1.3E‐03
	293.2124	5.506834	2	↓	−0.82	0.18	6.7E‐06	1.5E‐03
	291.1968	4.662	2	↓	−0.78	0.18	2.5E‐05	3.9E‐03
FA(22:4) isomer 2	331.2647	6.6415	2	↓	−0.77	0.19	4.7E‐05	4.6E‐03
FA(22:4) isomer 1	331.2649	6.610667	2	↓	−0.76	0.18	2.7E‐05	3.9E‐03
FAHFA(24:4)	391.2853	6.333167	1	↓	−0.75	0.18	3.2E‐05	4.0E‐03
EPA isomer 1	301.2173	6.240667	2	↓	−0.74	0.18	4.6E‐05	4.6E‐03
DHA isomer 1	327.2328	6.401	2	↓	−0.73	0.18	5.7E‐05	5.1E‐03
	323.2592	5.994	2	↓	−0.72	0.18	6.9E‐05	5.6E‐03
	347.26	4.93025	2	↓	−0.71	0.18	8.2E‐05	6.0E‐03
FA(22:4) isomer 3	331.2647	6.555167	2	↓	−0.70	0.18	1.7E‐04	0.01
HETrE	321.2408	5.365	2	↓	−0.69	0.18	1.2E‐04	8.5E‐03
	269.2124	5.173833	‐‐	‐‐	−0.68	0.18	1.6E‐04	0.01
	351.2183	3.545833	2	↓	−0.68	0.18	1.8E‐04	0.01
Arachidonic acid analog	303.2332	6.561333	2	↓	−0.68	0.18	2.1E‐04	0.01
Hydroxyoctadecanoic acid isomer 1	381.2625	5.365	2	↓	−0.65	0.18	2.9E‐04	0.01
Hydroxyoctadecanoic acid isomer 2	381.2626	5.4945	2	↓	−0.65	0.18	2.6E‐04	0.01
DHA isomer 2	327.233	6.357833	2	↓	−0.64	0.19	7.4E‐04	0.03
9(S) HOTrE	293.2122	3.971333	2	↓	−0.63	0.18	4.1E‐04	0.02
DHA analog	327.2332	6.524333	2	↓	−0.61	0.19	1.2E‐03	0.04
	299.2592	5.414333	2	↓	−0.61	0.18	7.9E‐04	0.03
Hydroxyhexadecanoic acid isomer 1	353.2318	4.0885	2	↓	−0.60	0.18	8.0E‐04	0.03
	299.2593	5.4945	2	↓	−0.59	0.18	1.0E‐03	0.04
Hydroxyhexadecanoic acid isomer 2	353.2314	4.6435	2	↓	−0.59	0.18	1.0E‐03	0.04
DHA isomer 3	327.2291	6.475	2	↓	−0.59	0.18	1.3E‐03	0.04
	269.2125	4.4585	2	↓	−0.59	0.18	1.4E‐03	0.04
	295.2281	5.038167	2	↓	−0.59	0.18	1.2E‐03	0.04
13‐oxoODE	293.2125	4.483167	2	↓	−0.59	0.18	1.5E‐03	0.04
5,6‐EET	319.2296	5.590083	1	↑	−0.59	0.18	1.2E‐03	0.04
	293.2125	5.0135	1	↑	−0.58	0.18	1.2E‐03	0.04
	326.2331	6.068	—	—	−0.58	0.18	1.8E‐03	0.04
	291.1967	4.711333	2	↓	−0.57	0.19	2.1E‐03	0.05
	295.228	5.1615	2	↓	−0.57	0.18	1.8E‐03	0.04
EPA isomer 2	301.2173	6.179	2	↓	−0.57	0.18	1.8E‐03	0.04
	299.2593	5.365	2	↓	−0.57	0.18	1.7E‐03	0.04
	317.2124	5.3465	2	↓	−0.55	0.18	1.8E‐03	0.04
	333.2073	3.2745	1	↑	0.67	0.19	4.3E‐04	0.02

*Note*: Beta estimates represent multivariable linear regression associations between metabolites and peak VO_2_. Models were adjusted for age, sex, BMI, aspirin use, statin use, and plate number. Significant values were defined as *q* < 0.05.

Abbreviations: 13‐oxoODE, 13‐keto‐9Z,11E‐octadecadienoic acid; 5,6‐EET: 5,6‐epoxyeicosatrienoic acid; 9(S) HOTrE, 9S‐hydroxy‐10E,12Z,15Z‐octadecatrienoic acid; DHA, 4Z,7Z,10Z,13Z,16Z,19Z‐docosahexaenoic acid; EPA, 5Z,8Z,11Z,14Z,17Z‐eicosapentaenoic acid; HETrE, hydroxyeicosatrienoic acid; m/z, mass‐to‐charge ratio.

Of the 39 metabolites with significant association between ∆MET and cardiorespiratory fitness, three (12.8%) displayed an acute increase with exercise (positive ∆MET). For two, greater magnitude of increase during acute exercise was associated with lower peak VO_2_. For instance, the AA epoxide derivative 5,6‐epoxyeicosatrienoic acid (5, 6‐EET) increased with acute exercise, and a greater magnitude of increase was associated with worse peak VO_2_ (β = −0.59, s.e. 0.18, *p* = 1.2 × 10^−3^). One metabolite with unknown molecular identity (m/z 333.2073, RT 3.2745) acutely increased with exercise, and a greater excursion was associated with greater peak VO_2_ (β = 0.67, s.e. 0.19, *p* = 4.3 × 10^−4^). In exploratory analyses we adjusted for peak RER as an additional covariate to account for exercise intensity and found that results were largely unchanged (Table S3).

### 3.3 Overlap of dynamic lipid metabolites associated with peak VO_2_ and other exercise traits

**FIGURE 3:**
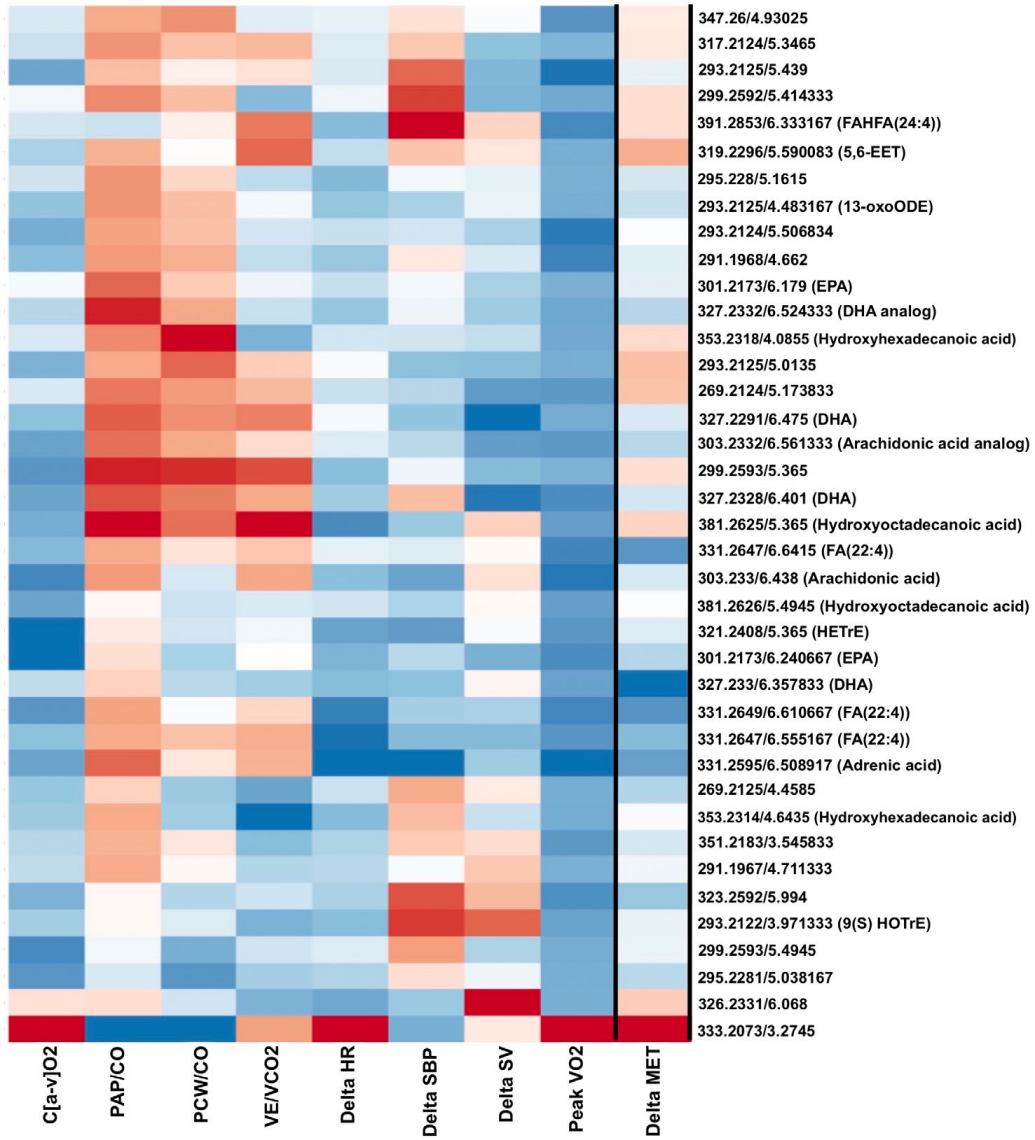
Overlap of 39 ∆MET associated with peak VO_2_ and other CPET parameters of exercise response, including change in blood pressure (∆SBP), heart rate (∆HR), stroke volume (∆SV), peripheral oxygen extraction (C[a‐v]O_2_), ventilatory efficiency (VE/VCO_2_ slope), diastolic reserve (PCWP/CO slope), and pulmonary vascular reserve (PAP/CO slope). Heat map shows absolute ∆MET (left column) with blue indicating decrease and red indicating increase with exercise and beta coefficients showing association of ∆MET with exercise traits, with positive associations shown in red and negative associations in blue. (heatmap created using MetaboAnalyst).

Of the 39 metabolites that demonstrated significant associations between ∆MET with peak VO_2_, the majority additionally demonstrated an overlap in significant associations with other exercise traits (Figure 3), including ∆HR (*n* = 14 [36%] metabolites) and peripheral oxygen extraction (∆C[a‐v]O_2_, *n* = 36 [92%], FDR *q* < 0.05 in multivariable‐adjusted analyses). By contrast, no significant associations (*q* < 0.05) were observed with ∆SBP, VE/VCO_2_ slope, or diastolic or pulmonary vascular reserve (PCWP/CO slope or PAP/CO slope; full results in Table S4). Top metabolites with known molecular identities associated with ∆HR included omega‐6 fatty acids and their derivatives including adrenic acid and HETrE, omega‐3 fatty acid EPA, and FA(22:4), and hydroxyoctadecanoic acid. For all of these, a greater decrease with exercise was associated with a greater heart rate response. Similarly, top lipid metabolites associated with C[a‐v]O_2_ include FA(22:4), hydroxyoctadecanoic acid, omega‐3 fatty acids EPA and DHA, and omega‐6 fatty acids and their derivatives including AA and its analog, adrenic acid, and DGLA derivative HETrE. Again, a greater decrease in metabolite with acute exercise was associated with greater peripheral oxygen extraction, consistent in directionality with overall fitness as measured by peak VO_2_. Of note, the pattern of associations of the 39 ∆MET with peak VO_2_ was most similar to the pattern of association of these ∆MET with C[a‐v]O_2_ (Figure S2).

### 3.4 Dynamic exercise metabolite clusters and clinical correlates

**FIGURE 4:**
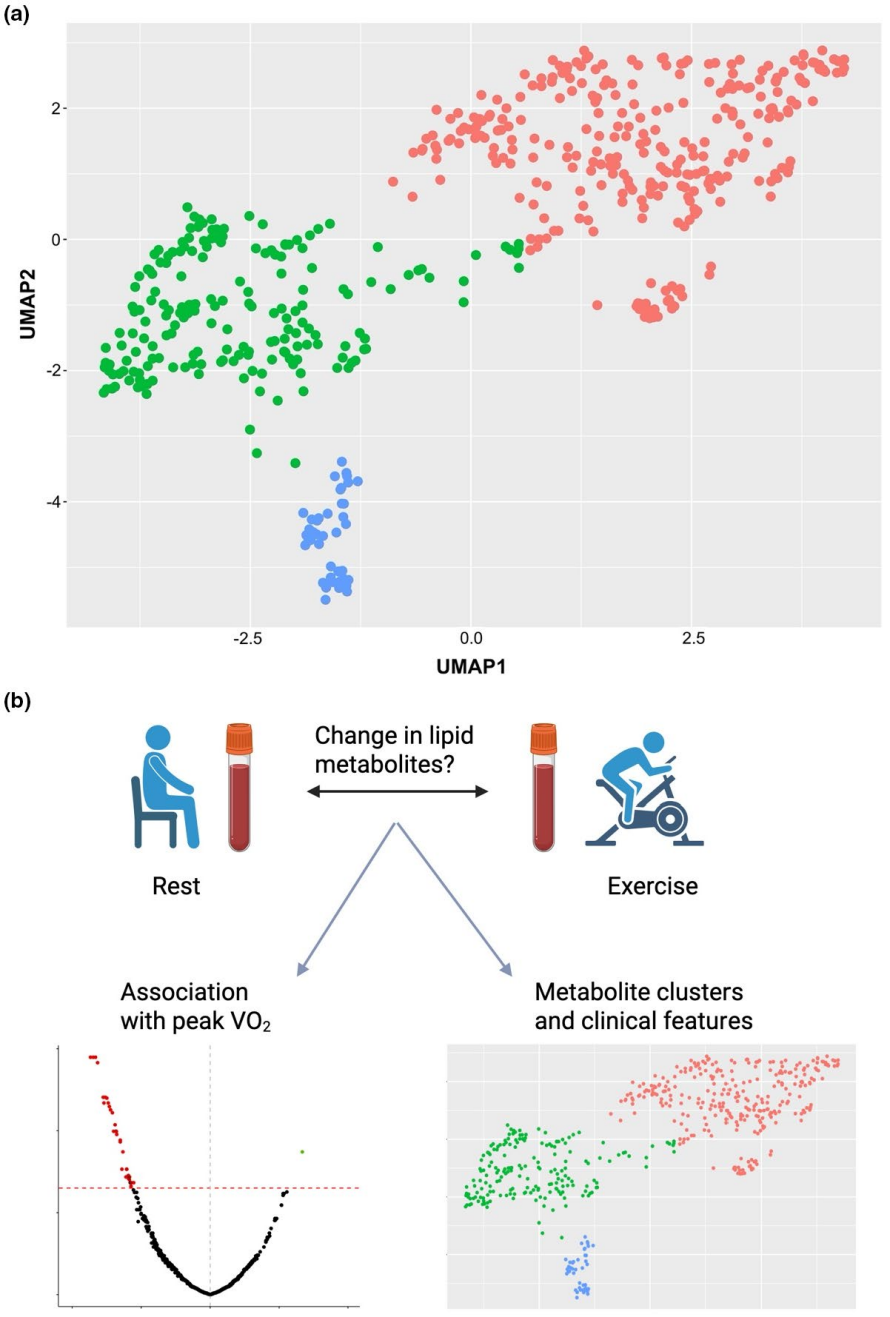
(a) UMAP of eicosanoids and related lipid metabolites that show significant acute exercise response. Metabolites cluster into three groups (red = Cluster 1, green = Cluster 2, blue = Cluster 3). (b) Schematic showing the process of identifying clusters within the metabolites with significant acute exercise responses.

In exploratory analyses, we performed UMAP of ∆MET to examine underlying patterns of exercise response and found three distinct clusters of ∆MET using hierarchical clustering (Figure 4a). These included 291 metabolites (cluster 1) that primarily increased with acute exercise, including linoleic acid derivatives 13‐HpOTrE and 9‐HpODE or 13‐HpODE, as well as AA derivatives 20‐carboxyarachidonic acid (20‐COOH‐AA), 11,12‐epoxyeicosatrienoic acid (11,12‐EET), and 15‐hydroperoxyeicosatetraenoic acid (15‐HpETE). There were 188 metabolites in cluster 2 that primarily decreased with exercise, including linoleic acid derivatives 6‐hydroxyoctadecadienoic acid (6‐HOME) and 13‐oxoODE, palmitic acid derivative hydroxyhexadecanoic acid, other omega‐6 fatty acids including AA and adrenic acid, omega‐3 fatty acids DHA and EPA, and FA(22:4). There were 44 metabolites in cluster 3 that on average decreased with greater magnitude after acute exercise compared with cluster 2, including oxononanoic acid or hydroxynonenoic acid, FA(18:2;O2), and linoleic acid derivatives including possibly dihydroxyoctadecadienoic acids (DiHODEs) and triHOMEs. (Figure S1).

To examine clinical characteristics across the three ∆MET clusters, we next calculated individual metabolite cluster scores and examined clinical correlates using partial correlation coefficients adjusted for age and sex (Table 4). Cluster 1 scores were positively associated with peak VO_2_ and C[a‐v]O_2_ and negatively associated with age and resistin (*p* < 0.01 for all), with weaker correlations with diabetes and leptin. Cluster 2 scores were positively associated with age and BMI and negatively associated with peak VO_2_ (*p* < 0.01 for all), with weaker correlations with C[a‐v]O_2_, leptin, and HOMA‐IR. Lastly, Cluster 3 scores were negatively associated with peak VO_2_ (*p* < 0.01), with weaker associations with BMI, diabetes, and HOMA‐IR (Table 4).

**TABLE 4:** Partial correlations of clinical variables with cluster scores.

Cluster score	1	2	3
Age	**−0.23 (3.5E‐07)**	**0.12 (5.7E‐03)**	0.09 (0.05)
Sex	−0.04 (0.37)	−0.02 (0.74)	−0.05 (0.24)
BMI	−0.08 (0.08)	**0.12 (6.6E‐03)**	**0.09 (0.04)**
DM	**−0.11 (0.02)**	0.07 (0.11)	**0.11 (0.02)**
HTN	−0.09 (0.06)	0.05 (0.24)	0.06 (0.22)
Peak VO_2_	**0.51 (7.3E‐34)**	**−0.14 (1.4E‐03)**	**−0.12 (7.0E‐03)**
C[a‐v]O_2_	**0.25 (1.2E‐08)**	**−0.10 (0.03)**	−0.07 (0.11)
HOMA IR	−0.07 (0.15)	**0.10 (0.03)**	**0.09 (0.04)**
CRP	−0.08 (0.07)	0.06 (0.20)	0.05 (0.31)
NT pro BNP	−0.07 (0.14)	−0.01 (0.79)	−0.001 (0.98)
Leptin	**−0.12 (0.01)**	**0.11 (0.02)**	0.08 (0.08)
Adiponectin	0.04 (0.40)	−0.06 (0.16)	−0.04 (0.44)
Resistin	**−0.13 (6.0E‐03)**	0.06 (0.19)	0.05 (0.27)
IL6	−0.07 (0.11)	0.09 (0.06)	0.08 (0.07)

*Note*: Values represent *r* (*p* value), where r values are partial correlation coefficients of cluster scores with clinical variables, adjusted for age and sex. Bolded values represent significant correlations, defined as *p* < 0.05.

Abbreviations: BMI, body mass index (kg/m^2^); CRP, C reactive protein; DM, diabetes mellitus; HOMA IR, homeostatic model assessment for insulin resistance; HTN, hypertension; IL6, interleukin 6; NT pro BNP, N‐terminal prohormone of brain natriuretic peptide.

## 4 DISCUSSION

In this study, we provide detailed molecular profiling of bioactive lipid and eicosanoid metabolites before and after an acute bout of exercise across a sample of nearly 500 individuals who underwent clinically indicated CPET. Our main findings are as follows: first, nearly 60% of assayed bioactive lipids displayed dynamic changes with acute exercise, half of which increased with exercise (including linoleic acid and AA derivatives), and half of which decreased (including triHOMEs and omega‐3 fatty acids). Further, we identified 39 metabolites for which the magnitude of acute change during exercise was also associated with peak VO_2_, a measure of overall cardiorespiratory fitness. Of these 39 metabolite associations, 37 (including omega‐3 and omega‐6 fatty acids and their derivatives and linoleic, palmitic, stearic, and AA derivatives) had overlapping associations with at least one other exercise trait, including chronotropic response and peripheral oxygen extraction. These findings not only highlight specific exercise lipokines involved in acute exercise, but also illuminate multiple molecular pathways as potential determinants of cardiorespiratory fitness.

Eicosanoid metabolites have previously been implicated as potential pro‐ and anti‐inflammatory mediators of cardiopulmonary disease (Lau et al., 2023; McNeill et al., 2023); however, the associations between the acute exercise responses of individual metabolites and measures of cardiorespiratory fitness remain incompletely understood. We identified 523 eicosanoids and related lipid metabolites that change dynamically with an acute bout of exercise. Specifically, metabolites that increased with acute exercise included hydroperoxide derivatives of linoleic acid involved in regulating oxidative stress and inflammation and AA derivatives with anti‐inflammatory properties known as specialized pro‐resolving mediators (SPMs) (Quehenberger & Dennis, 2011). These findings are consistent with previous studies that have shown that aerobic exercise can reduce serum levels of oxidants, lipid peroxides, and oxidative stress markers such as glutathione and increase levels of antioxidant agents such as nitric oxide and superoxide dismutase (Ye et al., 2021). We also identified the well‐studied lipid exerkine 12,13‐diHOME among the lipidome that increased with acute exercise and was associated with greater fitness, consistent with prior studies (Lynes et al., 2017; Pinckard et al., 2021; Robbins & Gerszten, 2023; Stanford et al., 2018). Further, our acute exercise results are mirrored by experimental studies demonstrating increased biosynthesis of SPMs in mice that underwent chronic exercise training (Calderin et al., 2022), though effects beyond an acute bout of exercise in humans remain to be studied.

We identified many molecules with dynamic increases during exercise which may implicate pathways involved in the regulation of inflammation, metabolism, and vascular function in cardiorespiratory fitness. Specific metabolites to highlight include 13‐HpOTrE, an alpha‐linoleic acid (ALA) metabolite which increased during acute exercise. Interestingly, 13‐HpOTrE has known anti‐inflammatory effects, with prior lipopolysaccharide‐induced inflammation models in cultured macrophage cells and mice demonstrating inactivation of the NLRP3 inflammasome complex, promoting apoptosis, and inhibiting autophagy in macrophages. (Kumar et al., 2016). Another metabolite that increased with exercise was 20‐COOH‐AA an oxylipin and AA metabolite of 20‐HETE that has been previously shown to modulate coronary vascular function, metabolism, and inflammation (Kaduce et al., 2004). Other studies have shown 20‐COOH‐AA to activate peroxisome proliferators‐activated receptor (PPAR) α and γ, modulating fatty acid and lipoprotein metabolism, as well as adipocyte differentiation, glucose metabolism, and inflammation (Fang et al., 2007). Furthermore, a study of blood oxylipin profiles of ST‐elevation myocardial infarction patients supports a role in modulating inflammatory and oxidative responses after cardiac ischemia and reperfusion (Solati et al., 2023). Overall, increases in these metabolites during exercise may implicate the role of physical activity in combatting inflammation and improving cardiometabolic and vascular health.

We also identified metabolites that displayed dynamic decreases during exercise, including prostaglandins, leukotrienes, and thromboxanes that play a role in inflammation, clotting, and smooth muscle contraction as well as fatty acids involved in membrane structure and function, lipid metabolism, and cell regulation. Other metabolites that decreased with exercise include linoleic and palmitic acid derivatives involved in inflammation and the oxidative stress response. Specifically, multiple triHOMEs, a class of oxylipin derivatives of linoleic acid, decreased during acute exercise (Fuchs et al., 2018). These metabolites have previously been found to be elevated across several lung diseases, including chronic obstructive pulmonary disease associated with neutrophilic abundance, as well as asthma (Balgoma et al., 2016; Lundström et al., 2012). These prior studies suggest that triHOMEs may exert pro‐inflammatory effects in lung parenchyma. Lastly, we noted that omega‐3 fatty acid derivatives of EPA and DHA also decreased with acute exercise, with exercise‐induced associated with higher peak VO_2_ in our study. Prior dietary supplementation studies of EPA and DHA have demonstrated benefits in chronic heart failure (Gissi‐Hf Investigators, 2008), and the recent REDUCE‐IT trial that demonstrated cardiovascular event risk reduction with the EPA derivative icosapent ethyl (Bhatt, 2019). However, prior studies of acute exercise changes in EPA and DHA derivatives have shown mixed results, with some indicating increased fatty acid oxidation and energy expenditure, whereas others show dependency on the type of exercise, that is, moderate‐intensity versus high‐intensity exercise. Thus, the effect of acute exercise on circulating levels of fatty acids including EPA and DHA remains to be fully delineated (Bahr et al., 1991; Phelain et al., 1997; Pritzlaff et al., 2000).

Next, we showed that for many bioactive lipids that demonstrate acute changes during exercise, the dynamic change with exercise in turn is associated with peak VO_2_ and other related exercise responses. For example, 13‐oxoODE decreases with acute exercise, and a greater decrease is associated with higher peak VO_2_, as well as better chronotropic response and peripheral oxygen extraction. An oxo metabolite of linoleic acid, 13‐oxoODE has been described to be a transcriptional activator binding to PPARγ, with known pro‐inflammatory and pro‐atherogenic effects, potentially indicating that the decrease in levels of 13‐oxoODE that we found to occur during acute exercise may be cardioprotective (Fang et al., 2007; Nagy et al., 1998; Tontonoz et al., 1998). Other metabolites associated with cardiorespiratory fitness include AA derivatives (5,6‐EET) and linoleic acid derivatives (9(S)‐HOTrE) with known anti‐inflammatory effects, hydroxylated fatty acids (FAHFA(24:4), hydroxyoctadecanoic acid, and hydroxyhexadecanoic acid), and AA derivatives (adrenic acid) and DGLA derivatives (HETrE) with mixed inflammatory effects. Taken together, these findings implicate the potential role of acute changes in anti‐inflammatory and oxidative stress response mediators during exercise in maintaining peak VO_2_, thereby suggesting a possible relationship between these pathways and cardiorespiratory fitness.

Of note, we found that the majority of dynamic lipid metabolites associated with peak VO_2_ were also associated with chronotropic response and peripheral oxygen extraction. By contrast, such overlap was absent for other hemodynamic exercise responses including ∆SBP, diastolic reserve (PCWP/CO slope), pulmonary vascular reserve (PAP/CO), and ventilatory efficiency slope (VE/VCO_2_). Our findings suggest that eicosanoids and related lipid metabolites may have more direct effects on peak VO_2_ via associations with peripheral oxygen extraction, which is highly dependent on vasodilation and vascular permeability in tissues (Schumacker & Samsel, 1989), whereas they bear less relevance to other exercise responses, which may be governed by a more diverse set of physiologic factors (ranging from ventricular relaxation to pulmonary vascular resistance) apart from those influenced by bioactive lipids.

To understand broader patterns of dynamic lipid metabolite responses to acute exercise, we used unsupervised hierarchical clustering to group metabolites into three distinct clusters. We found that cluster 1 was comprised of metabolites that increased with acute exercise, many of which had known cardioprotective functions including regulating and resolving inflammatory and oxidative stress cascades (e.g., 13‐HpOTrE, 20‐COOH‐AA) (Fang et al., 2007; Kaduce et al., 2004; Kumar et al., 2016; Solati et al., 2023). Further, higher cluster 1 scores and were associated with younger age, higher peak VO_2_, C[a‐v]O_2_, and lower resistin and leptin levels. By contrast, clusters 2 and 3 were comprised of metabolites that decreased with acute exercise and included metabolites known to be pro‐inflammatory and involved in lipid metabolism, oxidative stress, and cellular damage (e.g., 13‐oxoODE and triHOMEs) (Balgoma et al., 2016; Fang et al., 2007; Lundström et al., 2012; Nagy et al., 1998; Tontonoz et al., 1998). Cluster 2 and 3 scores were associated with older age, lower peak VO_2_, and greater cardiometabolic disease risk including higher BMI and insulin resistance. Taken together, these findings highlight cross‐sectional clinical and metabolic correlates that mark potential pro‐ versus anti‐inflammatory exercise metabolite responses relevant to fitness.

A number of limitations deserve mention. Given the observational and cross‐sectional nature of this study, causality cannot be determined; additionally, while we may derive useful insights as to the associations of specific metabolites with measures of cardiorespiratory fitness, the longitudinal effects of exercise training on bioactive lipids cannot be inferred. Second, our sample only included outpatients referred for clinical CPET in the setting of dyspnea, and generalizability to broader samples (i.e., to healthy populations without indication for CPET) is limited in that context. Third, while we adjusted for RER as a proxy for exercise intensity in analyses examining dynamic metabolite changes in relation to peak VO_2_, we acknowledge this may not fully account for potential differences in exercise duration and intensity. Fourth, metabolite profiling was performed using plasma samples. Different distributions of eicosanoids and lipid metabolites can be found in plasma versus serum, as serum can contain lipid metabolites released by activated platelets (Yasumoto et al., 2017). However, we used plasma samples to remain consistent with the original development and validation of the LC‐MS metabolite profiling method which also used plasma samples (Watrous et al., 2019). Fifth, plasma sample collection times were not recorded, and potential circadian rhythm‐related effects could not be evaluated. Lastly, this was a heterogeneous patient sample with comorbid medical conditions, many of which may affect bioactive lipid metabolism, with the potential for residual confounding.

## 5 CONCLUSIONS

In summary, we highlighted bioactive lipids and eicosanoids that underlie the metabolomic architecture of the acute exercise response, and we then showed direct associations of these metabolites with cardiorespiratory fitness. We found that pathways involving derivatives of linoleic and AA derivatives with known anti‐inflammatory actions increase with acute exercise, while pathways involving triHOME compounds and linoleic acid derivatives with pro‐inflammatory and pro‐atherogenic properties decrease with acute exercise. We also identified overlap among dynamic lipid metabolites associated with peak VO_2_ across other exercise traits, including chronotropic response, and peripheral oxygen extraction. Taken together, these findings provide a glimpse into dynamic metabolomic profiles of acute exercise and their relation to cardiorespiratory fitness. Future studies are needed to fully delineate metabolomic contributions to the salutary effects of exercise including chronic exercise training.

## FUNDING INFORMATION

JEH was supported by NIH grants R01‐HL140224, R01 HL160003, R01 HL168889, and K24 HL153669.

## CONFLICT OF INTEREST STATEMENT

J. Ho has received consultant fees from Eli Lilly. M. Jain currently holds equity and a leadership position at Sapient Bioanalytics, LLC, and is engaged in research related to the current study. All other authors have nothing to disclose.

## ETHICS STATEMENT

All participants provided written informed consent to participate, and the study protocol was approved by the institutional review boards of Beth Israel Deaconess Medical Center and Massachusetts General Hospital (protocol 2017P001587).

## Supporting information


Figure S1.

Figure S2.

Table S1.

Table S2.

Table S3.

Table S4.

## Data Availability

Raw LC‐MS mass spectrometry data files have been deposited in the UCSD data repository (https://massive.ucsd.edu/) under MSV000092775 and can be accessed at https://massive.ucsd.edu/ProteoSAFe/dataset.jsp?task=e17abc86a3254eb09d0147b4473369fc. The generated MGH CPET clinical data are considered sensitive patient data and can therefore not be publicly available in compliance with HIPAA and according to limitations included in the informed consents signed by the study participants. The datasets and code used during the current study are available from the corresponding author on reasonable request.
