# Supplementary material for: The role of bioactive lipids and eicosanoid metabolites in acute exercise in adults: Insights into human cardiorespiratory fitness
Source: Physiol Rep. 2025 Dec 5;13(23):e70671. doi: 10.14814/phy2.70671 (PMC12680789; doi:10.14814/phy2.70671)
Supplement: Supplementary file 1 — Figure S1. [file PHY2-13-e70671-s003.docx]

**S1 Figure:** Mean difference between peak exercise and rest in levels of metabolites that demonstrated a significant acute exercise response. Cluster 1 metabolites are shown in red, Cluster 2 metabolites in green, and Cluster 3 metabolites in blue.
