# Supplementary material for: The role of bioactive lipids and eicosanoid metabolites in acute exercise in adults: Insights into human cardiorespiratory fitness
Source: Physiol Rep. 2025 Dec 5;13(23):e70671. doi: 10.14814/phy2.70671 (PMC12680789; doi:10.14814/phy2.70671)
Supplement: Supplementary file 2 — Figure S2. [file PHY2-13-e70671-s001.docx]

**A)**

**B)**

**S2 Figure:** Relationships between the changes with acute exercise reflected by different CPET parameters, based on their associations with 39 ∆MET (as shown in Figure 3). **A)** Hierarchical clustering of the associations of these parameters with ∆MET. **B)** PCA vector plots of parameters with significant PCA loadings.
